# Aesthetic Surgical Crown Lengthening Procedure

**DOI:** 10.1155/2015/437412

**Published:** 2015-11-02

**Authors:** Pablo Santos de Oliveira, Fabio Chiarelli, José A. Rodrigues, Jamil A. Shibli, Vincenzo Luca Zizzari, Adriano Piattelli, Giovanna Iezzi, Vittoria Perrotti

**Affiliations:** ^1^Dental Research Division, Department of Periodontology, Guarulhos University, Guarulhos, SP, Brazil; ^2^College of Santa Teresa, Santa Teresa, ES, Brazil; ^3^Department of Medical, Oral and Biotechnological Sciences, University of Chieti-Pescara, Chieti, Italy

## Abstract

The aim of this case report was to describe the surgical sequence of crown lengthening to apically reposition the dentogingival complex, in addition to an esthetic restorative procedure. Many different causes can be responsible for short clinical crown. In these cases, the correct execution of a restorative or prosthetic rehabilitation requires an increasing of the crown length. According to the 2003 American Academy of Periodontology (Practice Profile Survey), crown lengthening is the most habitual surgical periodontal treatment.

## 1. Introduction

The common causes of short clinical crown include caries, erosion, tooth malformation, fracture, attrition, excessive tooth reduction, eruption disharmony, exostosis, and genetic variation [1]. Therefore, this deficiency in clinical crown length should be increased when margins of caries or margins of the tooth fractures are subgingivally placed, the crown is too short for retention of the restoration, there is an excess of gingiva, and anatomical tooth crown is partially erupted [2].

The ultimate goal of crown lengthening is to provide a tooth crown dimension adequate for a stable dentogingival complex and for the placement of a restorative margin, so as to achieve the best marginal seal and an aesthetically pleasing final restoration [3]. Several studies have also shown that a band of attached gingiva from 2 to 3 mm is preferable to successfully maintain the restored tooth. Since the resetting nature of this procedure, there is a risk of reducing the attached gingiva width; thus, this width should be carefully diagnosed and evaluated when planning crown lengthening procedure [4, 5].

Biologic width is defined as the physiologic dimension of the junctional epithelium and connective tissue attachment, according to the pioneering study conducted by Gargiulo et al. [6]. In this study, the authors demonstrated that humans, in average, show a connective tissue attachment of 1.07 mm, above the alveolar bone crest, and a junctional epithelium, below the base of the gingival sulcus, of 0.97 mm. The combination of these two measurements constitutes the biologic width, that is, 2.04 mm in average.

Ingber et al. suggested that an additional 1 mm might be coronally added to the 2 mm dentogingival junction, as an optimal distance between the bone crest and the margin of a restoration, to permit healing and proper restoration of the tooth [7]. In addition, during an esthetic crown lengthening procedure, bone removal plays an important role in the final location of the free gingival margin after healing.

Therefore, the aim of this case report was to describe the surgical sequence of crown lengthening to apically reposition the dentogingival complex, in addition to an esthetic restorative procedure.

## 2. Case Presentation

**Figure 1:**
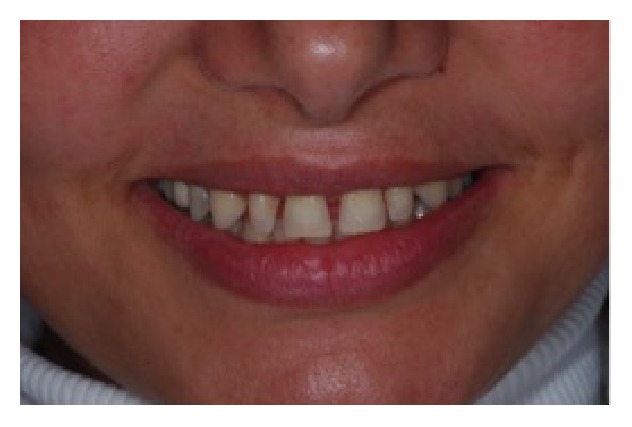
Clinical photograph representing the preoperative facial view with presence of diastemas.

**Figure 2:**
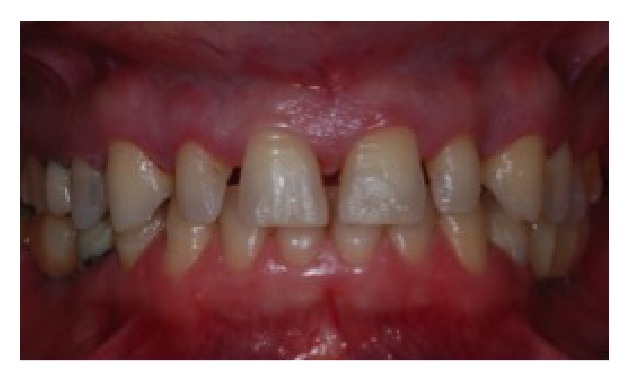
Preoperative intraoral view.

A 41-year-old woman was referred to the Department of Periodontology of a Private Clinic in Vila Velha, ES, Brazil. The patient presented a good general health and maxillary anterior teeth with short clinical crowns and diastemas (Figures 1 and 2). No periapical radiolucency at radiographic examination was detected, the periodontal ligament was within normal limit, and the crown-to-root ratio was about 1 : 3. At clinical examination, attached gingiva band was 6 to 7 mm in width, and periodontal pocket depth was 3 mm or less. Neither periodontal problems nor teeth mobility was detected. The primary concerns of this patient included anterior diastemas and dissatisfaction with the size and shape of teeth. The primary treatment plan proposed to the patient was an orthodontic option; however, the patient disagreed with this modality due to the wide duration time and financial burden. Therefore, the treatment plan realized was the crown lengthening of elements 13, 12, 11, 21, 22, and 23 and the installation of tooth veneers. The patient was informed about the treatment and a written consensus was obtained according to local legislation.

**Figure 3:**
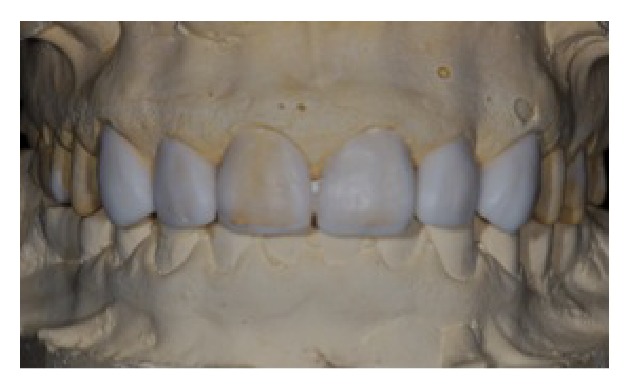
Diagnostic wax-up.

**Figure 4:**
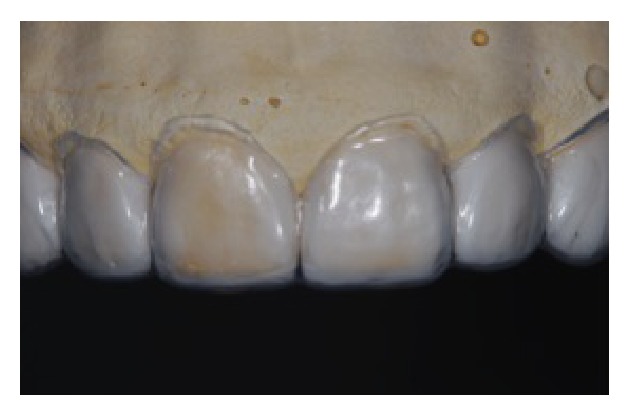
Surgical guide manufactured with silicone.

**Figure 5:**
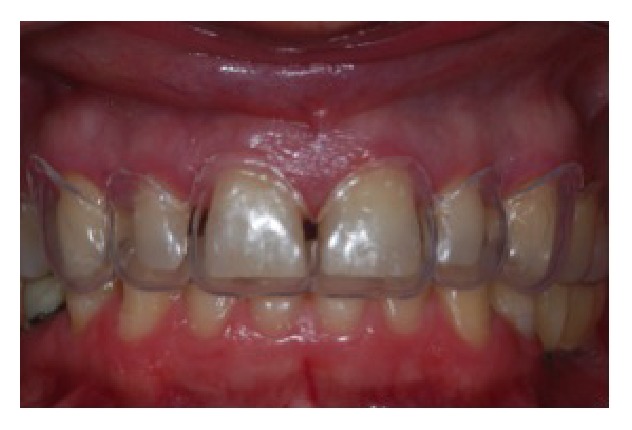
Surgical guide installed in the mouth.

**Figure 6:**
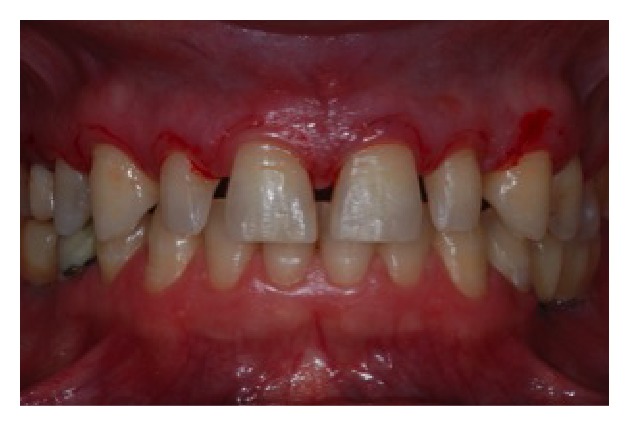
Register of new gingival margin with scalpel.

**Figure 7:**
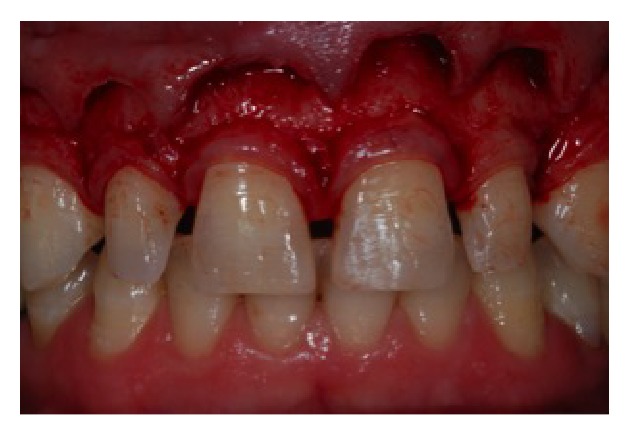
Incision in the registered area and elevation of a full-thickness mucoperiosteal flap.

**Figure 8:**
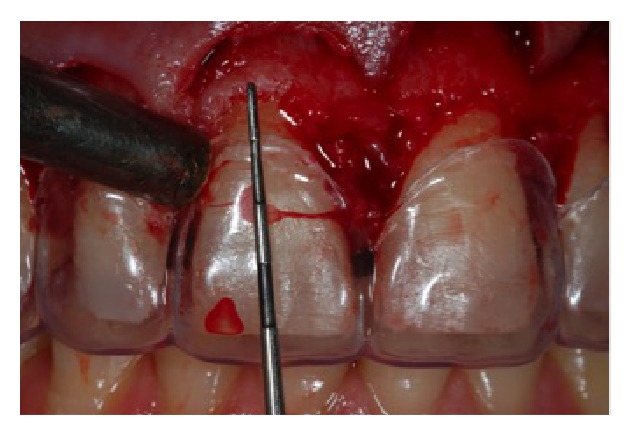
Measurements of the distance between the guide edge and the cervical bone for the osteotomy.

**Figure 9:**
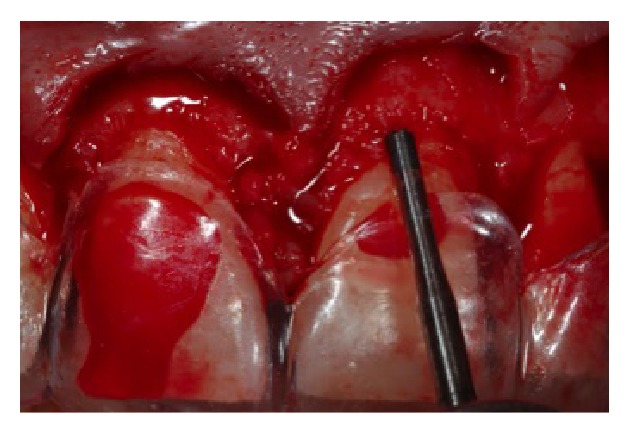
Removal of bone tissue with manual instrument.

**Figure 10 fig10:**
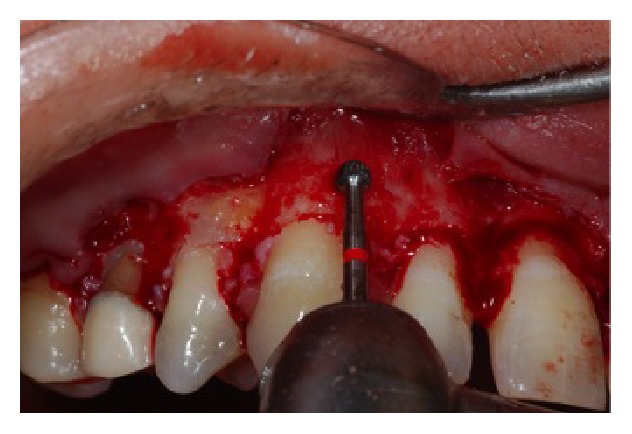
Bone tissue resection through rotating instrument.

**Figure 11:**
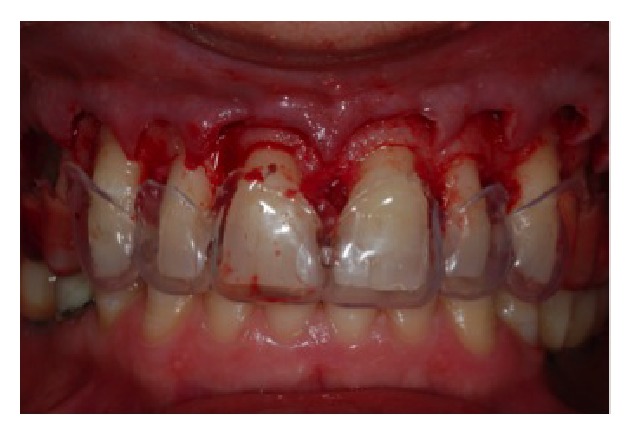
Clinical aspect after the bone removal.

**Figure 12:**
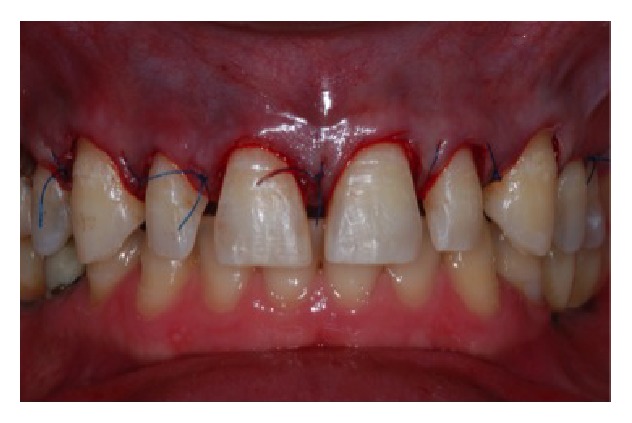
Suture of the surgical region.

**Figure 13:**
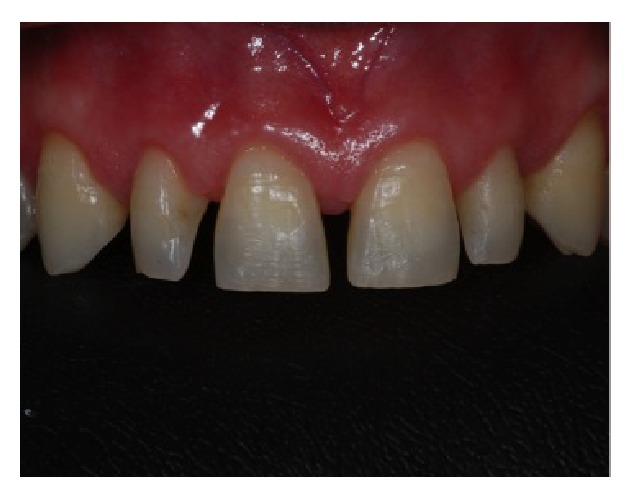
Clinical aspect of soft tissues after six-month follow-up.

**Figure 14:**
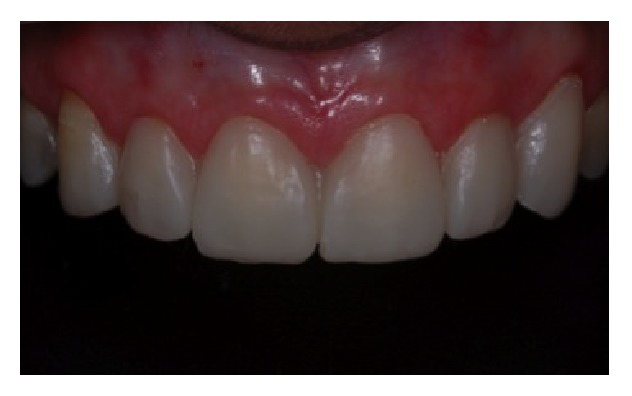
Installation of provisional facets.

Initially, an impression of the maxilla was obtained to realize the diagnosis wax-up (Figure 3), and then a surgical guide in silicone, with the edge tangent to the cervical region of wax-up, was confectioned (Figures 4 and 5). The guide was inserted in mouth and the new gingival margin was registered with a scalpel (Figure 6). Thus, a full-thickness mucoperiosteal flap was elevated (Figure 7) and the gingival collar extracted with a Gracey curette. For the osteotomy, measurement of the distance between the guide edge and the cervical bone was recorded (Figure 8). This distance should be about 3 mm, for the biologic width maintenance and installation of prosthesis. The creation of a precise biologic width requires, in addition, a precise osseous contouring, which was performed using manual instruments (surgical chisels) and carbide/diamond burs with adequate irrigation, for preventing bone necrosis (Figures 9 [Fig fig10]–11); then, the flaps were sutured (Figure 12). After 6-month healing (Figure 13), the provisional facets were installed for aesthetic test and posterior definitive prostheses were delivered (Figure 14).

## 3. Discussion

Crown lengthening is performed for aesthetic improvement during restorations and in teeth with subgingival caries or fractures; in addition, this surgical procedure can establish an accurate bone width [3] and correct gingival asymmetries [8].

The esthetic crown lengthening requires gingivectomy procedures to expose the needed additional tooth structure; therefore, a minimum of 2 to 5 mm of keratinized tissue is necessary to ensure the gingival health [9, 10]. Moreover, the management of the papilla is another important aspect of the surgery. The interproximal bone should be carefully removed in order to maintain the anatomic structures, so that the interproximal tissues are allowed to coronally proliferate; the papilla should replace the distance from the bone crest to the base of the contact area (about 5 mm or less) [11, 12]. Any smaller residual interproximal space can be eliminated by apically positioning the contact area of the definitive restoration [13, 14]. To have a harmonious and successfully long-term restoration, the distance between the crestal bone and prosthetic margins, which allows recreating the biological width, should be at least 3 mm [15]. This can be surgically achieved by crown lengthening, as presented in this case report, or orthodontically by forced tooth eruption or by a combination of both procedures [16].

Several studies suggest that the biologic width reestablishes itself after crown lengthening procedures, in 6 months [17–20]. For this reason, in the present case report the installation of definitive prosthesis was carried out after the healing period of the gingiva, in order to obtain the aesthetic position of the prosthetic margin.

In conclusion, crown lengthening surgery is a viable option for facilitating restorative therapy or improving esthetic appearance. However, to plan a crown lengthening procedure, the whole periodontal condition of the patients and their hygiene habits should be evaluated. Furthermore, an accurate diagnostic and interdisciplinary approach is mandatory for obtaining improved, conservative, and predictable results in esthetic areas.
